# Reactive Oxygen Species Are Central Mediators of Vascular Dysfunction and Hypertension Induced by Ethanol Consumption

**DOI:** 10.3390/antiox12101813

**Published:** 2023-09-29

**Authors:** Júlio C. Padovan, Thales M. H. Dourado, Gustavo F. Pimenta, Thiago Bruder-Nascimento, Carlos R. Tirapelli

**Affiliations:** 1 Laboratory of Blood and Vascular Biology, The Rockefeller University, New York, NY 10065, USA; padovaj@mail.rockefeller.edu; 2 Programa de Pós-Graduação em Farmacologia, Faculdade de Medicina de Ribeirão Preto, Universidade de São Paulo (USP), Ribeirão Preto 14040-902, SP, Brazil; thalesmhdourado97@usp.br (T.M.H.D.); gustavofelixpimenta@usp.br (G.F.P.); 3 Departamento de Enfermagem Psiquiátrica e Ciências Humanas, Laboratório de Farmacologia, Escola de Enfermagem de Ribeirão Preto, University of São Paulo, Ribeirão Preto 14040-902, SP, Brazil; 4 Department of Pediatrics and Vascular Medicine Institute (VMI), University of Pittsburgh, Pittsburgh, PA 15260, USA; bruder@pitt.edu

**Keywords:** oxidative stress, NADPH oxidase, ethanol, blood pressure, endothelium

## Abstract

Consumption of high amounts of ethanol is a risk factor for development of cardiovascular diseases such as arterial hypertension. The hypertensive state induced by ethanol is a complex multi-factorial event, and oxidative stress is a pathophysiological hallmark of vascular dysfunction associated with ethanol consumption. Increasing levels of reactive oxygen species (ROS) in the vasculature trigger important processes underlying vascular injury, including accumulation of intracellular Ca^2+^ ions, reduced bioavailability of nitric oxide (NO), activation of mitogen-activated protein kinases (MAPKs), endothelial dysfunction, and loss of the anticontractile effect of perivascular adipose tissue (PVAT). The enzyme nicotinamide adenine dinucleotide phosphate (NADPH) oxidase plays a central role in vascular ROS generation in response to ethanol. Activation of the renin–angiotensin–aldosterone system (RAAS) is an upstream mechanism which contributes to NADPH oxidase stimulation, overproduction of ROS, and vascular dysfunction. This review discusses the mechanisms of vascular dysfunction induced by ethanol, detailing the contribution of ROS to these processes. Data examining the association between neuroendocrine changes and vascular oxidative stress induced by ethanol are also reviewed and discussed. These issues are of paramount interest to public health as ethanol contributes to blood pressure elevation in the general population, and it is linked to cardiovascular conditions and diseases.

## 1. Introduction

The causal relation between heavy ethanol consumption and arterial hypertension was first established in 1915 by French army physician Camille Lian [1]. Although his findings were largely ignored for decades, Lian’s findings were eventually corroborated by several epidemiologic studies carried out in the late 1960s and in subsequent years in variable-size populations [2,3,4,5,6]. Attempts to evaluate a possible mechanism for ethanol-induced hypertension in humans were hindered by several limitations, including differences in type and frequency of ethanol consumption and variability in age, ethnicity, gender, body mass index, salt use, comorbidities (e.g., obesity, hypertension), and the use of medications. Studies conducted in the late 1970s and in the early 1980s provided evidence that heavy ethanol drinkers showed increased circulating levels of noradrenaline and renin, which suggested that ethanol promoted activation of both the sympathetic nervous system and the renin–angiotensin–aldosterone system (RAAS) [7,8,9,10]. However, these studies did not establish a causal relation between neuroendocrine changes and the hypertensive state induced by ethanol consumption. In the absence of a well-defined causality, experimental models of alcoholism became crucial to the understanding of the mechanisms underlying ethanol-induced hypertension.

Studies in animals substantiated the initial epidemiological findings in humans, confirming that ethanol consumption promoted neuroendocrine changes and led to increased blood pressures [11,12]. Moreover, those studies established a relationship between ethanol consumption and vascular dysfunction, suggesting the existence of a myogenic mechanism that might alter the contractile/relaxant properties of the vasculature, thereby contributing to the elevation of blood pressure. Current knowledge shows that chronic ethanol intake leads to functional and structural changes of the vasculature including endothelial dysfunction, inflammation, remodeling, and functional alterations, which are characterized by hypercontractility and impaired vasorelaxation [13]. Data support the idea that oxidative stress is a central mechanism whereby ethanol promotes its deleterious effects in the vasculature [14]. Distinctive vascular cell types can produce reactive oxygen species (ROS), and those include endothelial, smooth muscle and adventitial cells. In addition, immune system cells (e.g., neutrophils and macrophages) may also contribute to ROS generation in response to ethanol [15]

Described as the major source of ROS in the vasculature, the enzyme nicotinamide adenine dinucleotide phosphate (NADPH) oxidase plays a central role in the redox imbalance induced by ethanol in the vascular system [14]. This multi-subunit transmembrane enzyme consists of membrane and cytosolic subunits that relay specific information intracellularly in response to a wide variety of stimuli. In the vasculature, RAAS promotes activation and overexpression of NADPH oxidase through the action of angiotensin II and aldosterone, which further increases ROS production, leading to vascular dysfunction [16]. Ethanol increases both the activity and expression of some components of the NADPH oxidase, favoring overproduction of ROS. The redox imbalance induced by ethanol is further aggravated by decreased antioxidant responses [14].

This review discusses the mechanisms of vascular dysfunction induced by consumption of high amounts of ethanol, detailing the contribution of ROS to the pathobiology of ethanol in the vasculature. Data illustrating the association among neuroendocrine changes, ROS generation, and ethanol-induced vascular dysfunction are also addressed. A MEDLINE-based search was conducted using the keywords that follow: ethanol, alcohol, alcoholism, hypertension, blood pressure, vascular dysfunction, redox imbalance, reactive oxygen species, endothelial dysfunction, oxidative stress, NADPH oxidase, and antioxidant. In the next step, the titles, abstracts, and full article texts were screened and analyzed independently against our inclusion/exclusion criteria. Only articles published in English were considered. Reasons for the exclusion of articles include unclear ethanol dose or ingestion period. Amount, duration, and pattern of ethanol consumption influence the effects of this compound in the cardiovascular system. Here, we focus on the effects of chronic consumption of high amounts of ethanol.

## 2. Ethanol Consumption: A Risk Factor for Arterial Hypertension

In the 1970s, the Kaiser-Permanente Multiphasic Health Examination Data, from a landmark observational study, showed an increase of 11mmHg or more in systolic blood pressure in individuals consuming more than five drinks per day (one standard drink contains 14g of ethanol) in comparison to non-drinkers [2]. The study established a relationship between the amount of ethanol consumed and an increase in blood pressure, an observation that was subsequently corroborated by other reports [4,6], including the second Kaiser-Permanente study, published in 1986 [17]. Analysis of six prospective studies and twenty-nine cross-sectional studies in North America, Australia, Japan, Europe, and New Zealand, among other countries, showed that the association between arterial hypertension and ethanol consumption is independent of gender, age, body mass index, and smoking status. Prevalence of hypertension in individuals consuming 3–4 drinks/day is approximately 50% greater than that in non-drinkers, while in individuals that consumed 6–7 drinks/day the prevalence is 100% greater [1]. However, a specific population may itself be considered an important modifier of the prevalence of arterial hypertension induced by ethanol consumption. For example, it has been estimated that around 30% of the cases of arterial hypertension are attributed to ethanol in the United States and England [18], while in Australia the prevalence of hypertension attributed to ethanol consumption is 7% [19]; in comparison, prevalence was shown to be 24% in France [20].

Establishing a standard threshold for ethanol effects on blood pressure has been difficult and continues to be controversial. Whilst 1–2 drinks/day have been described to produce a slight or no effect on blood pressure [2,3,4,21], consumption of 3–5 drinks/day has been associated with increases in blood pressure, with more than 5 drinks/day promoting substantial increases in systolic blood pressure [2]. However, other studies have shown the threshold to occur at lower drinking levels [2,6]. In addition, regardingthe variations in the effect threshold, the extent of blood pressure increase due to ethanol consumption is also controversial. The Kaiser-Permanente Multiphasic Health Examination Data showed that more than 5 drinks/day promoted an increase of at least 11 mmHg in systolic blood pressure [2]. Other studies have shown that an acute intake of five drinks leads to increased values of systolic and diastolic blood pressures by 4–7 mmHg and 4–6 mmHg, respectively [22,23,24]. For chronic, albeit a comparatively more moderate, consumption of ethanol of 2–3 drinks/day for four weeks, average increases of 2.3 mmHg and 1.3mmHg have been reported for systolic and diastolic blood pressures, respectively [25]. A separate study conducted with chronic drinkers (≈4 drinks/day) for an average period of 21 years has shown that consumption of the same intake levels of four drinks/day of wine or beer leads to increases in systolic blood pressure of 2.9 and 1.9 mmHg, respectively [26].

Another important modifier of the ethanol–risk relationship for arterial hypertension is gender. It is well established that prevalence of arterial hypertension induced by ethanol consumption is lower in women than in men [2,17]. As described in the Risk Factor Prevalence Study [19], no more than 1% of hypertension cases in women were attributed to ethanol consumption. A systematic review and meta-analysis on the effects of ethanol consumption on blood pressure has revealed the existence of a J-shaped relationship for women, showing that consumption of one drink/day is associated with a reduced relative risk for hypertension, a somewhat protective effect, whereas 2drinks/day significantly increase the risk [27]. Similar findings have been described in another meta-analysis study that has also reported a J-shaped relationship for women [28]. More recently, it has been described that consumption of 1–2 drinks/day leads to increased risk of hypertension in male chronic drinkers in comparison to abstainers whereas no significant difference is observed between female chronic drinkers and corresponding abstainers [29].

Collectively, these studies have provided evidence that chronic consumption of high amounts of ethanol is an important risk factor for arterial hypertension development. However, available data for humans have not been sufficient to establish a substantial mechanism for blood pressure elevation as a result of ethanol consumption. The inherent limitations of epidemiologic studies in humans have been compensated by development of experimental models, which then corroborate the epidemiologic findings and confirm the pressor effects of ethanol. Additionally, the experimental studies showed a positive relationship between increases in blood pressure and the extent of ethanol consumption, describing the period of exposure to ethanol as an important factor in the development of arterial hypertension [30,31]. Blood ethanol concentration is another modifier of the relationship between ethanol consumption and hypertension. Based on experimental findings, it was proposed that higher levels of blood ethanol account for earlier development of arterial hypertension [30,32,33].

In 1983, in a pioneer study of the hypertensive effects of ethanol, Sutter et al. described a 25% increase in mean blood pressure (from 98 to 122 mmHg) in male Wistar rats treated with ethanol (20%v) for 12 weeks [11,34]. Subsequently, they showed that the ethanol-induced hypertensive state was accompanied by augmented levels of circulating noradrenaline [35], as had been observed in humans. Increased sympathetic activity and hypertensive states were also described in rats treated with ethanol (20%v) [30]. In ethanol-fed Sprague Dawley rats, blood pressures were higher at week 6 (from 106 to 147 mmHg), while an increase from 117 to 149 mmHg in blood pressure was observed in ethanol-fed Wistar rats only after eight weeks of treatment [30]. It is important to note that such changes in blood pressure were not the result of an upsurge but they rather had been gradually observed since the early stages of ethanol consumption. In some studies, for instance, blood pressure elevation was observed after four weeks of treatment with ethanol [31,36], whilst others evidenced this same response after only two weeks [33,37,38]. Differences in ethanol concentration in the blood may help explain the disparity among studies, since higher levels of blood ethanol were associated with earlier increases in blood pressure [36,39]. The effects of ethanol on blood pressure in experimental studies are summarized in Table 1.

Taking into consideration that elevated blood pressure is a strong predictor of impending/imminent cardiovascular diseases, hypertension associated with ethanol consumption may have important long-term clinical consequences. Some mechanisms have been postulated to explain the hypertensive response to chronic ethanol consumption: while human epidemiologic studies have proposed the involvement of a neuroendocrine mechanism, experimental studies have suggested the existence of a myogenic mechanism in which ethanol consumption promotes alterations in the vascular tonus. The latter is based on results obtained from studies describing that ethanol-induced hypertension occurs in parallel to changes in vascular responsiveness [31,36,39]. These mechanisms are discussed in the following sections.

## 3. Mechanisms Underlying Ethanol-Induced Hypertension: The Neuroendocrine and Myogenic Theories

### 3.1. Neuroendocrine Changes

A clear relationship between ethanol consumption and arterial hypertension had already been established by epidemiological studies by the end of the 1970s. It became important then to understand the mechanisms by which ethanol affected blood pressure. Studies in humans have attempted to address this question by evaluating the participation of the sympathetic nervous system and RAAS, two important regulators of blood pressure.

The contribution of the catecholamines noradrenaline and adrenaline to the pressor effects of ethanol was investigated in humans by measuring the circulating levels of these hormones, whose corresponding plasma concentrations were found to be higher after consumption of ethanol [10,53,54]. Based on those findings, it was proposed that the sympathetic nervous system mediates the prohypertensive effects of ethanol. However, other investigators showed that, despite blood pressure being higher among ethanol drinkers, no changes were detected in plasma levels of adrenaline or noradrenaline [6,22]. The reasons for such discrepancies are still uncertain, but they may be due to dose, frequency, and pattern of ethanol consumption. Experimental studies in animals corroborated the findings in humans, showing that consumption of ethanol increased both blood pressure and the circulating levels of the catecholamines [11,37]. However, neither the human studies nor the animal models managed to establish a causal relation between the activation of the sympathetic nervous system and the ethanol-induced increase in blood pressure. More recently, it was shown that ethanol promotes increases in both blood pressure and in the circulating levels of adrenaline and noradrenaline in male Wistar rats. Nebivolol (a selective β_1_-adrenergic receptor antagonist) prevented these responses, implicating a role of the sympathetic nervous system in the hypertensive state associated with ethanol consumption [40].

Regarding the participation of RAAS in ethanol-induced hypertension, pioneering works by many researchers revealed increases in the circulating levels of renin and aldosterone in heavy drinkers [7,8,55,56] and experimental studies strengthened the proposal that ethanol consumption activates RAAS. According to these data, ethanol increases plasma activity of renin and angiotensin-converting enzyme (ACE) and also increases the circulating levels of angiotensins I and II and aldosterone [37,41,57]. RAAS plays a critical role in mediating ethanol-induced hypertension since initial findings suggest that the increase in blood pressure induced by ethanol is mediated by angiotensin II [38]. Moreover, recent findings show the involvement of aldosterone in the hypertensive state induced by ethanol consumption [42]. The increase in blood pressure promoted by RAAS results from its effects in the vasculature and does not seem to be the result of changes in fluid and electrolyte balance [38,42].

Both the sympathetic nervous system and RAAS play a role in the pressor effects of ethanol and a relationship between these two systems to promote this response does exist. However, it is unknown if overactivation of those systems caused by ethanol intake are independent and work synergistically or if they communicate to each other in a positive feedback fashion. Ethanol-induced hypertension is accompanied by increases in the circulating levels of the catecholamines adrenaline and noradrenaline as well as by increased plasma levels of renin, angiotensin I, angiotensin II, and ACE activities. These responses are prevented by nebivolol, showing that β_1_receptors mediate RAAS activation. Thus, stimulation of β_1_-adrenergic receptor in juxtaglomerular cells seems to be the mechanism whereby ethanol indirectly (via catecholamines) triggers the release of renin leading to RAAS activation [40]. Importantly, AT_1_ receptor blockade with losartan prevents the increase in blood pressure induced by ethanol, implicating RAAS in this response [38].

Experimental and clinical findings also proposed that ethanol-induced neuroendocrine changes would promote increases in sodium handling, a mechanism that would explain the hypertensive state associated with ethanol. This hypothesis was stated based on findings showing that ethanol activated RAAS in humans, a response that occurred in parallel to increases in plasma levels of sodium [55,56]. Similarly, rats treated with ethanol showed increased circulating levels of sodium in parallel to RAAS activation [37]. However, a causal relation between RAAS activation and sodium handling to promote ethanol-induced hypertension remains unsettled.

Results from clinical and experimental studies led to the conclusion that the two major systems controlling blood pressure under physiological conditions, the sympathetic nervous system and RAAS, are implicated in the hypertensive state induced by ethanol and that there is an interplay between their corresponding responses. We discuss these findings further in Section 4.

### 3.2. The Myogenic Theory

Much of the experimental studies that investigated the effects of ethanol consumption on blood pressure also addressed the impact of the former on vascular responsiveness. Altogether, data from such reports revealed that ethanol both promotes vascular hypercontractility and impairs vascular relaxation in distinctive vascular territories.

Increased vascular reactivity to adrenoreceptor agonists has been described in the rat aorta and the carotid and mesenteric arteries [12,43,58,59,60,61]. The hypertensive state induced by ethanol is accompanied by increases in the pressor effects of intravenous phenylephrine (a selective α_1_-adrenoreceptor agonist) and endothelin-1 [33,44], supporting the concept that increased vascular responsiveness in vivo may be involved in the elevation of blood pressure induced by ethanol. Vascular hypercontractility induced by ethanol in the isolated aorta is endothelium-independent and maintained by two mechanisms: augmented production and release of thromboxane A_2_ (TXA_2_), a smooth muscle-derived vasoconstrictor prostanoid; and increased extracellular Ca^2+^ion influx [60]. In the aorta, increased TXA_2_ production is mediated by the cyclooxygenase (COX)-2 enzyme [42], but in resistance arteries, the mechanism whereby ethanol increases vasoconstriction in the presence of adrenergic agonists differs from that described in the aorta. In the mesenteric arterial bed, ethanol consumption induces an endothelium-dependent increase in phenylephrine-induced contraction. This response is the result of an augmented release of endothelial-derived vasoconstrictor prostanoids and an impaired modulatory action of endothelial nitric oxide (NO); the latter is likely associated with downregulation of the endothelial NO synthase enzyme (eNOS) [45].

The procontractile effect of ethanol is a complex and multi-mechanistic process. In the carotid artery, downregulation of ET_B_ receptors explains the hypercontractile effect to endothelin-1induced by ethanol. These receptors counteract the contraction induced by endothelin-1 by releasing NO [43,62]. In the microcirculation, ethanol consumption potentiates the vasocontractile effect of endothelin-1 by promoting upregulation of ET_A_ receptors, which mediate the contractile response induced by the peptide [63]. Overproduction of ROS is another mechanism by which ethanol promotes its vasocontractile effects. Drugs that display antioxidant effects are capable of preventing the procontractile effects of ethanol [40,46]. In fact, increased oxidative stress is a central mechanism whereby ethanol promotes vascular dysfunction, being responsible not only for the contractile effect but also for the impaired relaxation [14]. A more detailed overview on the contribution of oxidative stress to the vascular dysfunction induced by ethanol is provided in Section 5.

Impairment of vascular relaxation also accounts for the deleterious effects of ethanol in the vasculature. In the aorta, ethanol decreased the endothelium-dependent relaxation induced by acetylcholine [57]. Similar findings were described in resistance arteries in which the relaxation induced by these vasoactive agents was impaired after consumption of ethanol [44]. The NO-cyclic guanosine monophosphate (cGMP) pathway plays a prominent role in the vascular relaxation induced by acetylcholine [64], suggesting that the endothelial dysfunction is trigged by decreased NO bioavailability. In fact, the vascular expression of the enzyme eNOS as well as the levels of NO are reduced in the vasculature of ethanol-treated rats. These responses have a negative impact in the NO-cGMP pathway and are described to be mediated by ROS [47]. The participation of oxidative stress in the endothelial dysfunction induced by ethanol will be further discussed in Section 5.5.

Downregulation of endothelial ET_B_ receptors is also proposed as a mechanism whereby ethanol impairs vascular relaxation [62]. Activation of these receptors leads to the production of NO, and, consequently, ET_B_ receptors mediate vascular relaxation and counteract vascular contraction. Relaxation induced by IRL1620 (a selective endothelin ET_B_ receptor agonist), has been shown to be reduced in carotid arteries from ethanol-treated rats, while, as mentioned before, ethanol favors the contractile effect of endothelin-1 in carotid arteries [62]. In view of these observations, it is possible to conclude that downregulation of ET_B_ receptors figures as a mechanism impairing vascular relaxation and favoring vascular contraction.

Of importance, vascular mechanisms may be triggered to compensate for the blood pressure elevation induced by ethanol. For instance, it is known that the inducible endothelium-dependent hyperpolarizing factor (iEDHF) pathway is activated as a compensatory response to ethanol-induced hypercontractility. In rats chronically treated with ethanol, acetylcholine-induced relaxation of mesenteric arteries was significantly greater compared to control arteries and inhibitors of EDHF reversed this response [65].

Neuroendocrine changes followed by vascular hypercontractility are proposed as a sequence of events that explains the pathophysiological effects of ethanol in the cardiovascular system. In this scenario, final mediators of RAAS (angiotensin II and aldosterone) and the sympathetic nervous system (adrenaline and noradrenaline) would promote direct or indirect effects in the vasculature culminating in vascular hypercontractility and dysfunction. Interplay between these two mechanisms will be detailed in the next section.

## 4. Interplay of Neuroendocrine and Myogenic Changes in Mediating the Pressor Effects of Ethanol

RAAS is a major regulator of blood pressure under physiological conditions. Most of its actions, including sodium retention, aldosterone secretion (by the adrenal glands), and vasoconstriction, are mediated by angiotensin II, which exerts its biological actions through G-protein-coupled receptors AT_1_and AT_2_. Moreover, aldosterone stimulates sodium–water retention by acting on the kidney, leading to hypertension. Ethanol-induced hypertension correlates with elevated plasma angiotensin II levels, endothelial dysfunction, and impaired vascular relaxation [37,41]. Blockade of AT_1_ receptors prevents both the hypertensive state and the vascular hypercontractility induced by ethanol [38], showing the existence of a relationship between ethanol-induced RAAS activation and vascular changes. More recently, evidence on the participation of aldosterone in ethanol-induced vascular hypercontractility was provided [42]. Ethanol consumption increases the circulating levels of both angiotensin II and aldosterone [38,42]. The latter is a steroid hormone synthesized mainly by the adrenal glands and whose secretion occurs primarily in response to activation of AT_1_receptors by angiotensin II [66]. Blockade of AT_1_ receptors prevents ethanol-induced aldosterone release, showing that this response is mediated by angiotensin II [38]. The increase in blood pressure as well as vascular hypercontractility promoted by ethanol is abrogated by blockade of mineralocorticoid receptors [42], suggesting that aldosterone acts as the final mediator of RAAS to promote the pressor and procontractile effects of ethanol. Thus, the positive relationship between RAAS activation and vascular hypercontractility in response to ethanol consumption might help to explain the hypertensive effect of ethanol.

Production of angiotensin II also occurs locally within the vascular wall. Local RAAS may generate angiotensin II even when systemic RAAS is suppressed or normal. Under pathological conditions, vascular RAAS may amplify the effects of systemic RAAS, contributing to vascular oxidative stress and hypertension [67]. Ethanol consumption does not change the concentrations of angiotensin I or angiotensin II, nor does it alter the expression and activity of ACE in the vasculature. In addition, vascular expression of both AT_1_and AT_2_receptors is not affected by ethanol consumption, suggesting that, in spite of activating the systemic RAAS, ethanol does not activate the vascular angiotensin II-generating system [38].

**Figure 1:**
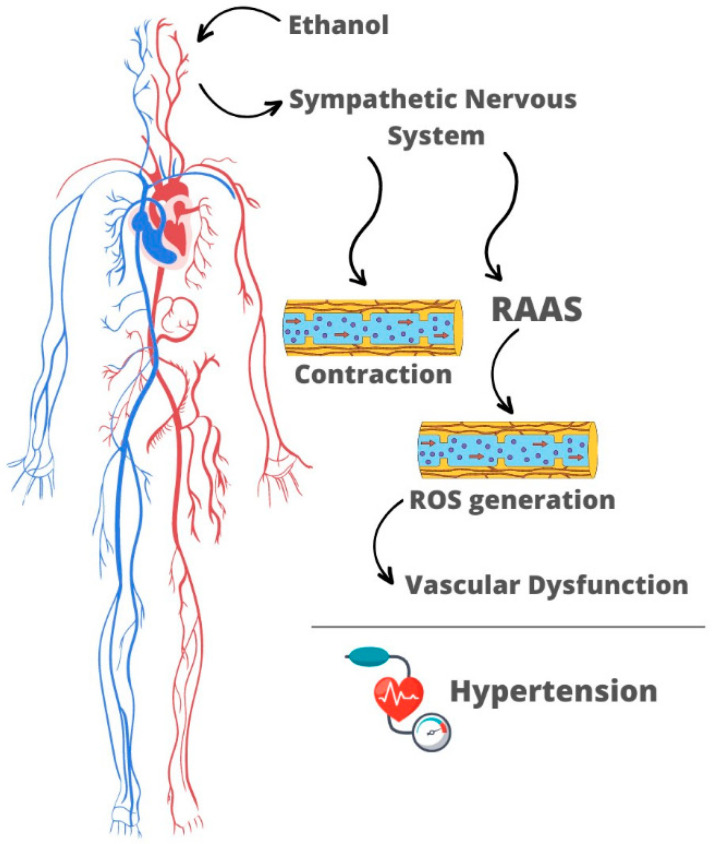
Proposal of the mechanisms underlying ethanol-induced hypertension. Neuroendocrine changes promoted by ethanol trigger overproduction of ROS in the vasculature, leading to vascular dysfunction. These responses culminate in hypercontractility and hypertension. RAAS: renin–angiotensin–aldosterone system; ROS: reactive oxygen species.

Ethanol-induced RAAS activation is proposed to be mediated by the sympathetic nervous system, since the hypertensive and procontractile effects of ethanol occur in parallel to increases in circulating levels of catecholamines [35,37] (Figure 1). The cardiovascular effects of ethanol involve a direct action of the sympathetic nervous system through activation of α- and β-adrenoreceptors [33,40]. Moreover, the sympathetic nervous system may trigger RAAS activation via β_1_-adrenergic receptors located in the juxtaglomerular cells, which synthesize, store, and secrete the enzyme renin. Blockade of β_1_-adrenergic receptors prevents an ethanol-promoted increase in the circulating levels of renin, angiotensin I, angiotensin II, and ACE activities, showing that the sympathetic nervous system modulates RAAS activation in response to the alcohol [40]. The central nervous system plays an important role in regulating sympathetic outflow and arterial pressure in response to ethanol consumption. It is proposed that an ethanol-induced increase in sympathetic outflow involves activation of NMDA receptors in the central nucleus of the amygdala neurons that projects to the rostral ventrolateral medulla [68].

Procontractile and hypertensive effects of ethanol result from the interplay between neuroendocrine and vascular changes. Ethanol centrally stimulates the sympathetic nervous system, which is responsible for triggering RAAS activation. The latter is critically involved in the vascular hypercontractility induced by ethanol consumption (Figure 1). Neuroendocrine changes promoted by ethanol trigger overproduction of ROS in the vasculature leading to disruption of the NO-cGMP pathway in the endothelium, thus favoring the accumulation of intracellular Ca^2+^ ions. These responses culminate in vascular dysfunction and hypertension.

## 5. Oxidative Stress: The Major Mediator of Vascular Dysfunction Induced by Ethanol

**Figure 2:**
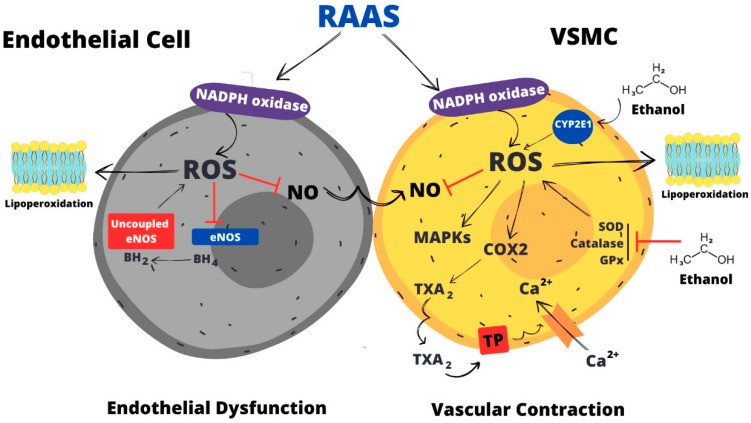
Summary of the molecular mechanisms underlying ethanol-induced vascular dysfunction. RAAS is an upstream mechanism that leads to NADPH oxidase activation, an enzyme that plays a central role in vascular ROS generation in response to ethanol consumption. Augmented levels of ROS trigger important processes underlying vascular injury including intracellular Ca^2+^ion accumulation, reduced NO bioavailability, MAPK activation, and endothelial dysfunction. VSMC: vascular smooth muscle cell; RAAS: renin–angiotensin–aldosterone system; ROS: reactive oxygen species; CYP2E1: cytochrome P450 2E1; COX2: cyclooxygenase 2; TXA_2_: thromboxane A_2_; MAPKs: mitogen-activated protein kinases; NO: nitric oxide; eNOS: endothelial NO synthase; BH_4_: tetrahydrobiopterin; BH_2_: dihydrobiopterin; TP: thromboxane receptor; SOD: superoxide dismutase; GPx: glutathione peroxidase.

Ethanol alters vascular tonus by disrupting the mechanisms that control and maintain the balance between contraction and relaxation. A number of possible mechanisms have been postulated to explain the pathogenesis of ethanol toxicity in the vasculature, which highlights the importance of identifying the biochemical/molecular basis of the ethanol effects (Figure 2). Overproduction of ROS is pointed out as a central mechanism whereby ethanol promotes vascular dysfunction and hypertension through increased generation of ROS and activation of redox-sensitive pathways, thereby reducing NO bioavailability and increasing intracellular Ca^2+^ion levels, actions that mediate the procontractile effects of ethanol.

### 5.1. NADPH Oxidase Is a Major Mediator of ROS Generation in Response to Ethanol

ROS stands for radical oxygen species produced either as intermediates or as a final reaction product. The vasculature produces superoxide anions (O_2_^•−^) and hydrogen peroxide (H_2_O_2_). These two compounds have important functions in the maintenance of vascular integrity and control of vascular tone by interacting with redox-sensitive target proteins under physiological conditions and activating redox-signaling pathways [69].

Oxidative stress is often described as a disturbance in the equilibrium established between ROS production and antioxidant defenses, which results in an increased bioavailability of ROS. Oxidative stress triggers pathophysiological processes including inflammation (by promoting platelet aggregation and monocyte migration), tissue hypertrophy, cellular proliferation, fibrosis, hypercontractility, and endothelial dysfunction, processes that are all involved in vascular dysfunction [70]. Thus, vascular dysfunction promoted by redox imbalance not only induces direct oxidative damage to macromolecules but also triggers redox signaling pathways in the vasculature that leads to changes in gene transcription and in oxidative modifications of proteins [71].

A number of enzymatic systems may generate O_2_^•−^ using oxygen as substrate, including xanthine oxidase, lipoxygenase, COX, uncoupled NO synthase (NOS), cytochrome P450 reductase, and some enzymes of the mitochondrial electron transport chain [72]. In these cases, ROS are generated as secondary products of the main chemical reactions catalyzed by those enzymes; however, for the enzyme NADPH oxidase, ROS consist of the main reaction product, with this enzyme being the major source of ROS in the vasculature, utilizing either NADPH or NADH as electron donors to promote the reduction of molecular oxygen into O_2_^•−^ [73]. The so-called “NOX family” is composed of seven isoforms of NADPH oxidase, which differ in their catalytic subunit (NOX1–5, DUOX1, DUOX2) and regulation. Whilst NOX1–4 are regulated by cytosolic proteins, NOX5 and DUOX1 and DUOX2 are activated by Ca^2+^ ions, which bind to specific domains located in these proteins [74]. In human vascular cells, the isoforms NOX1, NOX2, NOX4, and NOX5 are expressed and functionally active. NADPH oxidase-derived ROS play a role in both physiological and pathophysiological processes in the vasculature [73].

Overproduction of ROS by ethanol is crucial to its vascular pathophysiology. Similar to physiological conditions, O_2_^•−^ and H_2_O_2_are also central to the deleterious effects of ethanol in the vasculature, as well as in a variety of tissues, with enzymes of the NOX family playing a key role in the process [75,76,77,78]. Ethanol consumption has been described to promote increases in vascular NADPH oxidase activity, ROS generation, and lipoperoxidation. These responses are concomitant to changes in vascular tonus, which include hypercontractility and impaired vasorelaxation [41]. The augmented activity of NADPH oxidase promoted by ethanol may be associated with increased expression and phosphorylation of p47^phox^, a cytosolic protein that regulates the activation of NOX2 [48,79,80]. The effects of ethanol on NADPH oxidase also include increases in the expression of the catalytic subunits NOX1 and NOX2. This effect may help explain the augmented production of ROS in the vasculature promoted by ethanol consumption. In fact, NADPH oxidase is involved in other actions of ethanol including lipoperoxidation, vascular hypercontractility, and impaired vasorelaxation [46,47,81].

In the microcirculation, ethanol impairs vascular relaxation and increases NADPH oxidase activity, expression of NOX2, and translocation of p47^phox^, which is a crucial step for NOX2 activation [80]. Blockade of NADPH oxidase prevents the deleterious effects of ethanol in the microcirculation and attenuates the resulting increase in blood pressure, suggesting that the hypertensive state associated with ethanol consumption involves formation of ROS [47,49,80]. It is noteworthy that antioxidants are capable of preventing ethanol-induced overexpression of NOX1 and NOX2, implicating ROS in this response [40,46,80]. In this scenario, NADPH oxidase-derived ROS favor a positive feedback loop that amplifies the starting signal. Thus, the causal relationship between ethanol, ROS, and hypertension most likely occurs at the vascular level, where ethanol promotes activation/overexpression of NADPH oxidase, subsequently generating ROS, which are then implicated in vascular hypercontractility and impaired vasorelaxation. Altogether, these responses contribute to increases in vascular resistance and blood pressure.

NADPH oxidase activation is a multi-mediated and complex process since this enzyme responds to a wide range of stimuli. In the vasculature, mediators that control vascular tonus, such as endothelin-1, aldosterone, and angiotensin II, may promote NADPH activation [73]. The physiological actions of angiotensin II in the vasculature are predominantly mediated by AT_1_ receptors, which are also implicated in the pathophysiological actions of this peptide. Angiotensin II regulates the onset and progression of cardiovascular diseases by increasing NADPH oxidase activity and leading to upregulation of vascular NOX1 and NOX2, which are important in redox-mediated hypertension in various cardiovascular diseases [82]. The actions of angiotensin II on NADPH oxidase favor overproduction of O_2_^•−^, which, in turn, influence downstream signaling pathways. Angiotensin II figures as an important mediator of NADPH oxidase activation in response to ethanol. The actions of angiotensin II occur via AT_1_ receptors and include increased activity of NADPH oxidase and overproduction of ROS, responses that are directly implicated in the vascular hypercontractility and hypertension induced by ethanol [38].

There is also evidence showing the involvement of aldosterone in NADPH oxidase activation in response to ethanol. Aldosterone induces upregulation and increases in NADPH oxidase activity in the vasculature through activation of mineralocorticoid receptors [83,84]. The latter are implicated in the upregulation of NOX1 and in the increase in NADPH oxidase activity promoted by ethanol consumption in the vasculature. Activation of mineralocorticoid receptors leads to overproduction of ROS; the latter then mediate upregulation of COX2 and overproduction of the vasocontractile prostanoid TXA_2_, which ultimately induces vascular contraction [42].

NADPH oxidase is the major source of ROS in the vasculature and, consequently, this enzyme is implicated in the pathophysiology of multiple cardiovascular diseases [69]. Increased expression and activity of this oxidase is accepted as a central mechanism of the vascular effects promoted by ethanol [14]. NADPH oxidase-derived ROS are considered key factors in the development of endothelial dysfunction and hypercontractility induced by ethanol. Additionally, ethanol may lead to overproduction of ROS by activating targets other than NADPH oxidase. Other sources of ROS production in response to ethanol consumption are discussed in the next section.

### 5.2. Other Sources of Ethanol-Induced ROS Generation in the Vasculature

Ethanol-induced ROS generation may also occur through uncoupled eNOS and xanthine oxidase. In addition, ethanol metabolism is also associated with ROS production. However, the contribution of these sources to the deleterious effects of ethanol in the vasculature is not well determined.

The enzyme eNOS is a constitutive isoform of NOS present in the vasculature, where it promotes the generation of NO and L-citrulline using the amino acid L-arginine as substrate. The synthesis of NO occurs when eNOS is in its dimeric form, with L-arginine and the cofactor tetrahydrobiopterin (BH_4_) being crucial to its dimerization and activity. In its uncoupled form, eNOS may produce both O_2_^•−^ and NO, contributing to vascular dysfunction [85]. The reaction of these two molecules generates peroxynitrite (ONOO^−^), a very reactive free radical that oxidizes BH_4_, leading to eNOS uncoupling and subsequent generation of O_2_^•−^. Moreover, O_2_^•−^ is capable of oxidizing BH_4_, a process that also favors eNOS uncoupling [85]. A few studies assessed the role of uncoupled eNOS in ROS generation in response to ethanol. In this regard, ethanol consumption augments hepatic and renal concentrations of ONOO^−^.In the vasculature, ethanol consumption induced increased staining for nitro tyrosine, suggesting the production of high levels of ONOO^−^ [40]. Ethanol consumption promotes BH_4_deficiency, which occurs in parallel to an impaired eNOS-dependent vasodilatation [86,87]. An in-vivo study showed that, in the microcirculation, ethanol consumption compromises endothelium-dependent vasorelaxation and this response is reversed by administration of BH_4_, suggesting a role for uncoupled eNOS in this response [88]. This is indirect evidence that suggests a possible contribution of uncoupled eNOS to ROS generation induced by ethanol.

Xanthine oxidase contributes to O_2_^•−^ generation in conditions such as hypertension and coronary arterial disease [89]. By promoting the reduction of xanthine or hypoxanthine to uric acid, xanthine oxidase also reduces one or two electrons of molecular oxygen leading to the generation of O_2_^•−^ or H_2_O_2_ as intermediaries [90]. During the reduction of xanthine or hypoxanthine, one atom of hydrogen is transferred from these substrates to NAD generating NADH. Ethanol may influence xanthine oxidase activity by promoting an imbalance of the NAD/NADH ratio during its oxidation [91]. In the liver, ethanol was described to promote an increase in lipid peroxidation that was mediated by xanthine oxidase [92]. Despite playing a role in the hepatic effects of ethanol, the participation of xanthine oxidase in the vascular effects of ethanol remains elusive.

The central route whereby ethanol is metabolized in the liver takes place in the cytosol where the enzyme alcohol dehydrogenase converts ethanol into acetaldehyde, which is then oxidized to acetate in the mitochondria by the enzyme acetaldehyde dehydrogenase. The conversion of ethanol into acetaldehyde may also occur in the peroxisome and microsome by the enzymes catalase and cytochrome P450 2E1 (CYP2E1), respectively. Ethanol metabolism by the CYP2E1 pathway produces ROS. This response may be amplified when ethanol is chronically consumed since in this case it induces the expression of CYP2E1.In addition, ethanol is metabolized by alcohol dehydrogenase and CYP2E1 in extrahepatic tissues, the sympathetic nervous system, and RAAS [93]. ROS derived from ethanol metabolism react with macromolecules (e.g., nucleophilic proteins, phospholipids, and nucleic acids) and also activate intracellular pathways that leads to tissue inflammation and apoptosis.

Both alcohol dehydrogenase and CYP2E1 are functionally active in the vasculature [94]. Ethanol promotes direct actions in the vasculature where it induces both catalytic activity and expression of ethanol-metabolizing enzymes. These responses occur in parallel with overproduction of ROS. The increase in oxidative stress induced by ethanol leads to activation of the myosin light chain (MLC) kinase, subsequent phosphorylation of MLC and tight-junction proteins, decreased blood-brain barrier integrity, and increased monocyte migration across blood-brain barrier [94,95]. Thus, vascular metabolism of ethanol leads to generation of ROS that will, in turn, affect vascular integrity. In addition, inhibition of the ethanol-metabolizing enzymes dehydrogenase and CYP2E1 reduces the direct vasomotor effects exerted by ethanol, showing that ethanol metabolism in the vasculature influences vascular tonus.

### 5.3. Impairment of Antioxidant Systems May Contribute to Ethanol-Induced ROS Accumulation

Cellular levels of ROS are regulated by enzymatic and non-enzymatic antioxidant systems. While the enzymes superoxide dismutase (SOD), catalase, and glutathione peroxidase constitute the most important enzymatic systems, ascorbate, tocopherols, glutathione, bilirubin, and uric acid are pointed out as the major non-enzymatic antioxidants [96]. Enzymes of the enzymatic antioxidant system are expressed on blood vessels, where they play an important role in the control of redox balance.

There are three isoforms of SOD: the cytoplasmic Cu/Zn-SOD or SOD1, the mitochondrial Mn-SOD or SOD2, and the extracellular Cu/Zn-SOD or SOD3; all of which are expressed in the vasculature. These enzymes promote the dismutation of O_2_^•−^ in H_2_O_2_ and oxygen in distinctive intracellular compartments [97]. Since O_2_^•−^ is a highly unstable and reactive molecule, enzymes of the SOD family are considered as the first line of defense against free radicals. The reaction of these antioxidant enzymes with O_2_^•−^ maintains the physiological levels of O_2_^•−^, preventing cellular damage. Ethanol can diminish SOD activity. For example, heavy drinkers (≥6 drinks/day) show decreased plasma SOD activity when compared to abstainers [98]. Similar findings are described in an animal model of ethanol consumption [40]. Experimental findings showed that ethanol decreased total SOD activity in the vasculature [99]. Activity of both Cu/Zn-SOD and Mn-SOD were depressed in the vasculature of ethanol-treated rats, a response that was related to decreased NO bioavailability and endothelial dysfunction. The exact mechanism whereby ethanol diminishes SOD activity is unknown, but this response is proposed to be mediated by ONOO^−^ [41]. In the microcirculation, ethanol consumption reduces SOD activity and SOD1 expression, these effects being attributed to ROS [80].

H_2_O_2_ is a stable and membrane-permeable ROS which is involved in the activation of distinctive redox signaling pathways. H_2_O_2_displays mild oxidant properties and for this reason it is inert to most biomolecules. In fact, H_2_O_2_contributes to the physiological regulation of the vascular tone by promoting activation of potassium channels and increasing the generation of NO production [100]. However, H_2_O_2_promotes alterations of amino acid residues (e.g., cysteinyl residues) located in active or allosteric sites of some proteins, leading to modifications of their activity and function. Phosphatases, transcription factors, ion channels, antioxidant enzymes, structural proteins, and protein kinases are examples of proteins that may be modified by H_2_O_2_ [101].

Among those proteins, a group of protein kinases named mitogen-activated protein kinases (MAPKs) is of special interest. MAPKs belong to four families of proteins (the extracellular signal-regulated kinase 1/2 (ERK1/2), p38MAPK, jun *N*-terminal kinase (JNK), and the extracellular signal-regulated kinase 5 (ERK5)) that are key components of signaling pathways. While proteins of the ERK cascade play a role in proliferation, differentiation, growth, and cell survival, JNK and p38MAPK are involved in apoptosis/inflammation and inflammatory responses, respectively [101]. Since all MAPKs are targets of H_2_O_2_, cellular consequences derived from their redox regulation by H_2_O_2_are ample.

The concentration of H_2_O_2_is regulated by intracellular and extracellular enzymes, such as catalase, which converts H_2_O_2_into H_2_O and O_2_. Since catalase is a key enzyme in the metabolism of H_2_O_2_, decreases in its expression and/or activity may result in increased H_2_O_2_bioavailability [102]. Ethanol consumption decreases vascular catalase activity, but the consequences of this response are unknown [41,99]. Diminished catalase activity may favor an increase in H_2_O_2_concentration in vascular cells, leading to redox regulation of signaling pathways. In fact, studies in isolated arteries showed that ethanol consumption is linked to MAPK activation in the vasculature. Inhibitors of p38MAPK and ERK1/2 attenuate ethanol-induced contraction and increase in intracellular Ca^2+^ ions, showing the involvement of these proteins in the vasocontractile response to ethanol [103,104]. In vitro studies in isolated arteries showed that ethanol consumption increases vascular p38MAPK and SAPK/JNK phosphorylation as well as expression of SAPK/JNK, responses that occur in parallel to increased NADPH oxidase-derived ROS and vascular hypercontractility [38,46,47,79]. Thus, MAPKs contribute to the vascular pathobiology of ethanol, but the involvement of H_2_O_2_in their activation needs further clarification.

The effects of ethanol on catalase activity are dependent on the amount and frequency of consumption and amount of ethanol consumed. Some reports have shown that ethanol may increase catalase activity in the vasculature, leading to a decreased bioavailability of H_2_O_2_ [49,80,81]. The increase in catalase activity in response to ethanol was also described in other tissues as a compensatory mechanism to protect against the deleterious effects displayed by H_2_O_2_ [80,105]. At this point, it is important to reiterate that the vascular effects of H_2_O_2_are concentration-dependent. At low concentrations, H_2_O_2_exerts vasorelaxation, while high concentrations of H_2_O_2_are associated with vasoconstriction [100]. Thus, ethanol-induced increases in catalase activity in the vasculature may favor H_2_O_2_-mediated vascular relaxation. In the vasculature, an ethanol-induced increase in catalase activity leads to a decrease in H_2_O_2_concentration [49,80], but the possible contribution of H_2_O_2_ to vasorelaxation in such conditions remains to be determined. Conversely, ethanol-induced decreases in catalase activity may favor the vasocontractile action of H_2_O_2_.

Glutathione peroxidase promotes the reduction of H_2_O_2_to H_2_O and catalyzes the conversion of lipid hydroperoxides to their corresponding alcohols. Eight isoforms of glutathione peroxidase are currently described (glutathione peroxidases1–8). These enzymes vary in cellular location and substrate specificity. They work with SOD and catalase, forming an enzymatic antioxidant system that promotes ROS reduction, limiting their cellular toxicity. In order to reduce H_2_O_2_ and alkyl hydroperoxides, all members of the glutathione peroxidase family use glutathione (GSH) as substrate, a reducing agent that is converted to glutathione disulfide (GSSG), its oxidized form during the reduction process [106]. Clinical and experimental studies revealed that ethanol consumption promotes reduction in the activity of glutathione peroxidase in serum [50,98,107]. In the vasculature, ethanol induces decreases in glutathione peroxidase activity, GSH levels, and of the GSH/GSSG ratio [41,81,99]. The possible contribution of these responses to the deleterious effects of ethanol in the vasculature is unknown. As described for catalase, a decrease in glutathione peroxidase activity may favor an increase inH_2_O_2_, which acts as a signaling second messenger in the vasculature triggering multiple signaling pathways involved in vascular dysfunction.

Increased levels of ROS may be the result of an imbalance between their generation and elimination by antioxidant systems. Ethanol promotes a negative regulation of antioxidant enzymes and this response may contribute to vascular increases in ROS levels induced by ethanol.

### 5.4. Ethanol-Induced Oxidative Stress Leads to Ca^2+^Ion Accumulation in the Vasculature

Ca^2+^ions are essential for smooth muscle contraction. Increases in intracellular Ca^2+^ion concentration during excitation may occur due to Ca^2+^ion release from intracellular stores (sarcoplasmic reticulum or mitochondria) or extracellular Ca^2+^ion influx through voltage- or ligand-gated ion channels located in the cell membrane. The increase in intracellular Ca^2+^ions in smooth muscle cells is one mechanism by which ethanol consumption promotes vascular hypercontractility. In-vivo and in-vitro studies provided evidence that ethanol augments vascular concentration of Ca^2+^ions by promoting increases in Ca^2+^ion uptake in smooth muscle cells [51,108]. Of importance, ethanol-induced intracellular Ca^2+^ion accumulation is associated with vascular hypercontractility [60,109].

Redox-sensitive signaling pathways mediate the contraction and increase in intracellular Ca^2+^ions induced by ethanol in vascular smooth muscle cells. In vitro, antioxidants attenuate both the elevation in intracellular Ca^2+^ions and the vasocontractile effect induced by ethanol, implicating ROS in such responses [110,111]. Ethanol effects on Ca^2+^ion accumulation are mediated by H_2_O_2_and O_2_^•−^, which trigger production of COX-derived vasoconstrictor prostanoids (prostaglandin F_2α_ and TXA_2_) that ultimately increases intracellular Ca^2+^ion concentration in vascular smooth muscle cells and their contraction [112] (Figure 2).

In vivo, ethanol consumption promoted elevations in vascular Ca^2+^ion influx and this response occurred in parallel to hypertension [51,52,113]. In vitro, the increase in Ca^2+^ion influx mediated by ethanol occurs through voltage-sensitive channels and is linked to the procontractile effect exerted by ethanol [114]. As in studies in vitro, ROS are also implicated in the ability of ethanol to promote accumulation of Ca^2+^ionsin vivo. The vascular hypercontractility associated with ethanol consumption is mediated by TXA_2_, a vasoconstrictor prostanoid that stimulates Ca^2+^ion influx through the cell membrane [60]. In isolated vessels, it was shown that the increase in TXA_2_production is mediated by the proinflammatory enzyme COX2, whose vascular expression is induced by ethanol. ROS are implicated in the upregulation of COX2, overproduction of TXA_2_,and vascular hypercontractility as their production is regulated by aldosterone [42]. Thus, the RAAS, through the action of aldosterone, triggers vascular hypercontractility with ethanol. By activating mineralocorticoid receptors, aldosterone induces ROS generation, which will in turn induce upregulation of COX2 and overproduction of TXA_2_. The latter will ultimately stimulate extracellular Ca^2+^ion influx leading to vascular contraction (Figure 2).

A role for ROS in Ca^2+^ion mobilization and vasoconstriction induced by ethanol is well established. Overproduction of ROS, Ca^2+^ion influx, and vascular contraction are interrelated and might contribute to ethanol-induced hypertension.

### 5.5. Role of ROS in Endothelial Dysfunction Induced by Ethanol

The vascular endothelium produces NO, a relaxing factor that plays a key role in the control of vascular tone. NO is a free radical generated from the amino acid L-arginine by the action of the three isoforms of the enzyme NOS. While neuronal NOS (nNOS or NOS1) and endothelial NOS (eNOS/NOS3) are the isoforms constitutively expressed in the vasculature, the inducible NOS (iNOS/NOS2) isoform is expressed in response to inflammatory stimuli. In the vascular endothelium, eNOS is the main isoform responsible for NO production. Once synthesized in the endothelium, NO diffuses to vascular smooth muscle cells where it stimulates the soluble isoform of guanylyl cyclase (sGC), an enzyme that catalyzes the synthesis of cGMP from guanosine 5′-triphosphate (GTP). cGMP activates protein kinase G (PKG) that, acting by multiple mechanisms, will induce smooth muscle relaxation [115].

Endothelial dysfunction is a systemic pathological state of the endothelium characterized by a decrease in NO bioavailability and activation of the NO-cGMP pathway. In general, reduced NO bioavailability may be the result of a decreased production of NO by endothelial eNOS or, more frequently, an increased breakdown by O_2_^•−^, which reacts with NO leading to the generation of ONOO^−^. Endothelial dysfunction is triggered by different cardiovascular risk factors such as hypertension, obesity, and diabetes [116]. Ethanol consumption induces overproduction of ROS in the vasculature and for this reason is considered a risk factor for endothelial dysfunction.

Endothelial dysfunction is assessed in vitro by evaluating endothelium-dependent vasorelaxation induced by vasoactive substances (e.g., acetylcholine) that stimulate endothelial release of NO. This pharmacological approach has been widely used to evaluate the impacts of ethanol consumption in endothelial function. In vitro studies using isolated arteries showed that ethanol consumption decreases the endothelium-dependent relaxation induced by acetylcholine, a response that is mediated by endothelial-derived NO [117]. The impairment in endothelium-dependent vasorelaxation promoted by ethanol is related to a decreased production of NO by eNOS [118,119]. In fact, ethanol consumption reduces eNOS expression and this response occurs in parallel to a decrease in acetylcholine-induced relaxation [45]. In cultured endothelial cells and isolated blood vessels, antioxidants prevented the downregulation of eNOS as well as the decrease in NO bioavailability and impairment in acetylcholine-dependent relaxation, implicating ROS in such responses [47,87,120]. Thus, ROS mediate endothelial dysfunction in response to ethanol by downregulating eNOS and by inducing NO inactivation. NADPH oxidase is implicated in the production of endothelial ROS that will further promote endothelial dysfunction induced by ethanol. Inhibition of this enzyme restored the impairment of endothelium-dependent relaxation induced by ethanol consumption. Endothelial dysfunction mediated by NADPH oxidase-derived ROS is linked to both impaired vascular relaxation and hypertension induced by ethanol consumption. As discussed in Section 5.1, ethanol-induced activation and expression of NADPH oxidase in the vasculature are mediated by the RAAS [38,42].

Ethanol consumption also compromises the synthesis of NO by interfering with BH_4_, a cofactor that is necessary for eNOS during the synthesis of NO. In vitro findings in isolated arterioles showed that by decreasing BH_4_, ethanol consumption impairs endothelium-dependent dilatation [87]. Reduced metabolism of BH_4_leads to eNOS uncoupling resulting in an increased generation of O_2_^•−^ and a reduced production of NO [121]. In vivo findings showed that ethanol consumption promotes a decrease in arteriolar flow-induced vasodilation. The microvascular dysfunction is restored by BH_4_administration, showing that reduction of the eNOS cofactor has a negative impact on NO production. In addition, it may be concluded that uncoupled eNOS may contribute to the impaired vasorelaxation of the microcirculation induced by ethanol [122]. Ethanol-induced decreased availability or utilization of BH_4_favors O_2_^•−^ generation resulting in an imbalance between O_2_^•−^ and NO, thereby contributing to endothelial dysfunction, presumably by NO inactivation [87] (Figure 2).

Ethanol metabolism in the vasculature may play a role in the endothelial dysfunction induced by ethanol consumption. ADH and CYP2E1 are ethanol-metabolizing enzymes that are constitutively expressed and functionally active in the vasculature. CYP2E1 promotes the conversion of ethanol into acetaldehyde, but this process leads to the generation of O_2_^•−^ [94]. The latter reacts with NO, decreasing its bioavailability, a response that can account for the impaired endothelium-dependent relaxation promoted by ethanol consumption [79,112].

Endothelial dysfunction induced by ethanol may be aggravated by overexpression of iNOS, the inducible isoform of NOS [81,114]. This enzyme induces a substantial and sustained release of NO that readily reacts with O_2_^•−^, forming ONOO^−^, an oxidizing molecule that is linked to endothelial dysfunction. In the microcirculation, induction of iNOS expression by ethanol is associated with a decrease in NO bioavailability and impaired endothelium-dependent relaxation. These responses occurred in parallel to overproduction of O_2_^•−^ and were prevented by the antioxidant apocynin, showing that ethanol-induced iNOS upregulation is mediated by ROS [80].

Under physiological conditions, endothelium-derived NO counteracts vascular contraction. In this sense, endothelial dysfunction not only compromises vascular relaxation but also favors vascular contraction. Decreased endothelial modulation of the vascular contraction was described after ethanol consumption. In all cases, this response was a consequence of reduced NO bioavailability and occurred in parallel to impaired endothelium-dependent vascular relaxation [12,45,62].

Endothelial function may also be assessed in vivo using the non-invasive method of brachial artery flow-mediated dilation (FMD) [123]. This method is widely used to determine endothelium- and NO-mediated vasodilatation in vivo in experimental and clinical studies. Lower values of brachial artery FMD are linked to a higher risk of future cardiovascular events. Individuals with a history of chronic alcoholism (≥6 drinks/day for ≥2 years) or with a history of repeated binge drinking show decreased brachial artery FMD when compared to non-drinkers [124,125,126,127]. Reduced endothelial function induced by heavy ethanol consumption may predispose individuals to future cardiovascular diseases, including hypertension [122].

A decrease in NO bioavailability is a central mechanism whereby ethanol promotes endothelial dysfunction (Figure 2). This response results from prejudiced activation or downregulation of eNOS, responses that are mediated by ROS. In addition, O_2_^•−^ generated by NADPH oxidase, uncoupled eNOS, or ethanol metabolism reacts with NO, reducing its bioavailability and resulting in impaired vasorelaxation. Endothelial dysfunction impairs vasorelaxation and represents an important mechanism underlying the effects of ethanol on blood pressure.

### 5.6. Perivascular Adipose Tissue (PVAT) and Its Role in Ethanol-Induced ROS Production

PVAT is a complex tissue composed predominantly of adipocytes, but other cell types including mesenchymal stem cells and immune cells are also found in PVAT. It surrounds most blood vessels and displays phenotypic heterogeneity depending on the vascular territory. While PVAT surrounding the thoracic aorta has a brown adipose tissue-like phenotype, PVAT that surrounds the abdominal aorta and coronary arteries is a mixture of white and brown adipose-like tissues. Conversely, mesenteric, femoral, and carotid arteries are surrounded by PVAT that is predominantly composed of white adipose-like tissue [128,129].

PVAT displays an anticontractile effect by releasing a wide range of vasoactive substances, such as NO, H_2_S, H_2_O_2_, prostacyclin, palmitic acid methyl ester, and angiotensins1–7. The contribution of each one of these substances to the regulation of vascular tone is dependent on PVAT composition (brown-like or white-like adipocytes) and, for this reason, varies according to the vascular bed. The anticontractile phenotype of PVAT is seen under physiological conditions, but it may shift to a procontractile one under certain pathophysiological circumstances, such as hypertension and obesity [130]. The procontractile effects of PVAT are mediated by decreased production/release of anticontractile substances and increased generation of procontractile factors, such as O_2_^•−^, angiotensin II, noradrenaline, prostaglandins, and chemerin [129].

There are a few reports describing the impact exerted by ethanol exposure in PVAT. Current data show that the effects of ethanol in PVAT vary according to the vascular territory and pattern of ethanol consumption. PVAT counteracts the procontractile effect induced by a single dose of ethanol. In this scenario, the vascular protective effect of PVAT is the result of a decreased activity of catalase that favors an increase in H_2_O_2_ concentration. In this case, the anticontractile effect displayed by PVAT-derived H_2_O_2_is partially mediated by NO [131].

In periaortic PVAT, long-term ethanol consumption increases the production of ROS via NADPH oxidase activation. As a consequence, there is a reduction in NO bioavailability in PVAT. Despite inducing molecular changes, ethanol does not favor a procontractile phenotype of periaortic PVAT or induce loss of its anticontractile effect [15]. However, chronic ethanol consumption favors a procontractile phenotype of PVAT that surrounds mesenteric arteries. This response is mediated by the proinflammatory cytokine IL-6, whose concentration is augmented in plasma and PVAT after ethanol consumption. The procontractile phenotype induced by IL-6 involves activation of NADPH oxidase in PVAT, with further increases in O_2_^•−^ generation. IL-6 derived from PVAT also mediates intravascular recruitment of neutrophils in response to ethanol, showing that PVAT may shift to a proinflammatory phenotype in response to ethanol [15].

**Figure 3:**
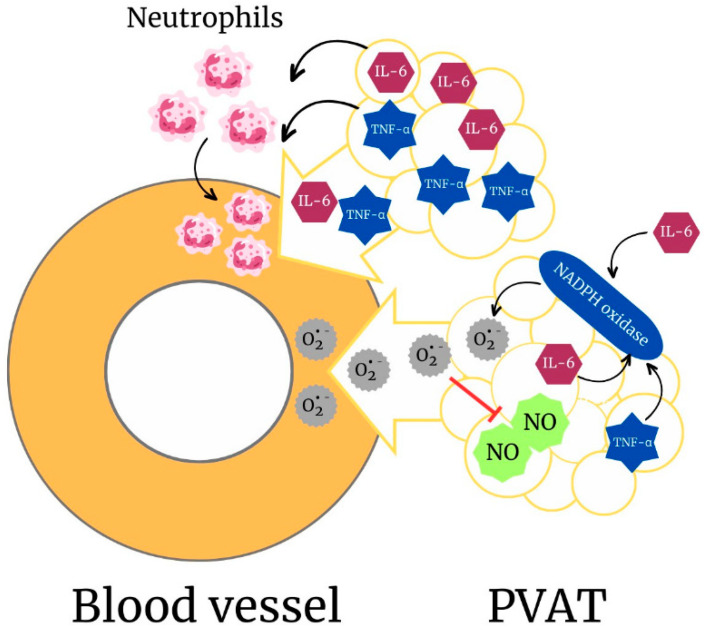
Ethanol consumption promotes dysfunction of PVAT. Ethanol consumption favors a proinflammatory phenotype of PVAT by promoting increases in the levels of IL-6 and TNF-α. These cytokines are linked to activation of NADPH oxidase, ROS generation, and decreased bioavailability of NO. PVAT: perivascular adipose tissue; O_2_^•−^: superoxide anion; NO: nitric oxide.

So far, studies support the notion that PVAT may be a target of the effects of ethanol, while it also contributes to the deleterious effects displayed by ethanol in the vasculature (Figure 3). PVAT is a metabolically active organ that under non-physiological conditions contributes critically to cardiovascular disease onset and progression. In this scenario, dissecting the precise role of PVAT in the vascular effects of ethanol is of paramount interest.

## 6. Direct Effects of Ethanol in the Vasculature 

Ethanol exerts direct effects in the vasculature. At low concentrations, ethanol induces relaxation while at higher concentrations it promotes vasoconstriction. Pioneer studies of Dr. Altura, using in-situ and in-vitro approaches, have shown that ethanol inhibits vasoconstriction induced by distinctive vasoactive agents and promotes concentration-dependent vasorelaxant actions on rat venules, veins, arterioles, and arteries [132,133,134,135]. Inhibition of Ca^2+^ ion uptake by vascular smooth muscle cells was initially suggested as a mechanism whereby low concentrations of ethanol (170–430 mmol/L) promoted relaxation [136]. Functional studies in the rat aorta and mesenteric arterial bed additionally showed that ethanol attenuated both extracellular and intracellular Ca^2+^ ion mobilization in vascular smooth muscle [137].

The effect of ethanol on Ca^2+^ion mobilization is not the only mechanism associated with ethanol-induced direct vasorelaxation. Functional studies provided evidence that endothelial-derived factors including NO [138,139] and prostaglandin [138] play a role in ethanol-induced vasorelaxation. In cultured bovine aortic endothelial cells and human umbilical endothelial cells, ethanol increased both mRNA and protein expression of eNOS as well as the concentration of NO [140]. Ethanol (10 and 50 mmol/L) also stimulated NO production in cultured umbilical vein endothelial cells via stimulation of Ca^2+^ ion-activated potassium channels [141]. This evidence shows that vascular relaxation induced by ethanol involves the participation of both endothelial and vascular smooth muscle cells [137,138].

We provided evidence that redox-sensitive and NO-dependent signaling pathways mediate ethanol-induced vascular relaxation [142]. Based on data obtained in functional assays and in cultured vascular smooth muscle cells from rat aortas, we showed that the endothelium-independent vasorelaxant action of ethanol (0.03–200 mmol/L) is mediated by the NO-cGMP pathway and also involves the opening of both ATP-sensitive and voltage-dependent K^+^ion channels. Production of NO in response to ethanol occurs rapidly by the activation of a constitutive isoform of NOS. Antioxidant treatment of endothelium-intact or -denuded aortic rings prevented ethanol-induced relaxation, showing that this response is mediated, in part, by ROS. Ethanol metabolism by ADH and CYP2E1 in the vasculature is possibly the main source of ROS generation, which trigger the vascular production of NO leading to vascular relaxation [142].

The direct vasocontractile effect of ethanol occurs at concentrations higher than those needed to promote relaxation. Initial studies in this field showed that ethanol (8–570 mmol/L) induced a concentration-dependent response in endothelium-intact or endothe-lium-denuded basilar and middle cerebral arteries, from dogs, sheep, piglets, and baboons. In all cases, ethanol-induced contraction was dependent of extracellular and intracellular Ca^2+^ ion mobilization [143]. Exposure of cultured smooth muscle cells for seven days to crescent concentrations of ethanol of 46, 115, and 460 mg/dl led to increases in cytosolic free Ca^2+^ions of 22%, 56%, and 58%, respectively [108]. Importantly, ethanol-induced increases in cytosolic free Ca^2+^ions in cultured smooth muscle cells as well as ethanol-induced contraction of cerebral arteries were attenuated by the antioxidant α-tocopherol, suggesting a role for ROS in such responses [110,144]. Later, it was demonstrated that H_2_O_2_ modulates the increase in cytosolic free Ca^2+^ions induced by ethanol, implicating this ROS in ethanol-induced vascular contraction [111].

A more detailed description of the direct vasocontractile effects of ethanol was provided by Yogi et al. [112]. Ethanol was found to induce contraction of isolated rat aortic rings with intact or denuded endothelium, but endothelial cells counteract ethanol-induced contraction via activation of the NO-cGMP pathway. Ethanol increased H_2_O_2_concentration in cultured vascular smooth muscle cells and functional assays revealed that catalase, a scavenger of H_2_O_2_, blunted the vascular contraction induced by ethanol, supporting a role for H_2_O_2_in this response. Their findings also revealed that the vasocontractile prostanoids PGF_2α_and TXA_2_ derived from COX1 are involved in ethanol-induced contraction. In addition, ROS were demonstrated to be involved in prostanoid-mediated contraction. Finally, it was described that O_2_^•−^ and COX1-derived metabolites are important mediators of ethanol-induced increases in intracellular Ca^2+^ ions. Collectively, the findings of Yogi et al. [112] supported the proposal that ROS and COX pathways are associated with ethanol-induced smooth muscle contraction and increases in intracellular Ca^2+^ ions.

Altogether, the studies designed to evaluate the direct vascular action of ethanol showed that its effects are concentration-dependent. In addition, neither contraction nor relaxation is dependent on the presence of the endothelium. These studies identified signaling pathways whereby ethanol promotes vasoconstriction and vasorelaxation. Although these results shed light on putative molecular mechanisms whereby ethanol mediates direct actions in the vasculature, we must be aware of the fact that they cannot be necessarily extrapolated to in vivo conditions. 

## 7. Conclusions

Ethanol consumption is associated with a robust oxidative response in the vasculature, which correlates to hypertension and neuroendocrine changes. The latter are characterized by increased sympathetic activity and activation of RAAS, an upstream mechanism that contributes to NADPH oxidase activation, overproduction of ROS and vascular dysfunction. NADPH oxidase-derived ROS trigger important processes underlying vascular injury including intracellular Ca^2+^ ion accumulation, reduced NO bioavailability, MAPK activation, endothelial dysfunction, and loss of the anticontractile effect of PVAT. Redox imbalance induced by ethanol in the vasculature leads to impaired vasodilatation and hypercontractility, which are recognized as central events in the hypertensive state induced by ethanol consumption. Thus, it is of great importance to invest in implementing strategies that help to prevent alcoholism, reducing the risk of ethanol-associated cardiovascular diseases.

## Figures and Tables

**Table 1:** Summary of the effects of ethanol on blood pressure in experimental models.

Species *	Dose/Period of Treatment with Ethanol	Increase in Blood Pressure
Wistar rats	Ethanol 20%v/12 weeks	25% in MAP (from 98 to 122 mmHg) [11]
Wistar rats	Ethanol 20%v/12 weeks	27% in SBP (from 117 to 149 mmHg) [30]
SpragueDawley rats	Ethanol 20%v/12 weeks	38% in SBP (from 106 to 147 mmHg) [30]
SpragueDawley rats	Ethanol 20%v/22 weeks	26% in SBP (from 115 to 145 mmHg) [31]
Wistar rats	Ethanol 20%v/13 weeks	21% in MAP (from 98 to 119 mmHg) [33]
Wistar rats	Ethanol 20%v/12 weeks	21% in MAP (from 102 to 124 mmHg) [34]
Wistar rats	Ethanol 20%v/12 weeks	24% in MAP (from 98 to 122 mmHg) [35]
Wistar rats	Ethanol 7.2%v/4 weeks	12% in SBP (from 112 to 125 mmHg) [36]
Wistar rats	Ethanol 20%v/4 weeks	19% in MAP (from 98 to 117 mmHg) [37]
Wistar rats	Ethanol 20%v/5 weeks	16% in SBP (from 125 to 144 mmHg) [38]
Wistar rats	Ethanol 20%v/5 weeks	17% in SBP (from 119 to 140 mmHg) [40]
Fisher rats	4g/kg/12 weeks	52% in MAP (from 92 to 140 mmHg) [41]
Wistar Hannover rats	Ethanol 20%v/5 weeks	29% in MAP (from 92 to 119 mmHg) [42]
Wistar rats	Ethanol 20%v/9 weeks	25% in MAP (from 97 to 122 mmHg) [43]
Wistar rats	Ethanol 20%v/5 weeks	23% in MAP (from 85 to 105 mmHg) [44]
Wistar rats	Ethanol 20%v/9 weeks	23% in MAP (from 98 to 121 mmHg) [45]
Wistar rats	Ethanol 20%v/5 weeks	13% in SBP (from 124 to 140 mmHg) [46]
Wistar Hannover rats	Ethanol 20%v/9 weeks	25% in SBP (from 126 to 158 mmHg) [47]
Fisher rats	4g/kg/12 weeks	42% in MAP (from 98 to 140 mmHg) [48]
C57BL/6 mouse	Ethanol 20%v/12 weeks	15% in SBP (from 115 to 132 mmHg) [49]
Fisher rats	4g/kg/12 weeks	54% in MAP (from 90 to 142 mmHg) [50]
WistarKyoto rats	Ethanol 10%v/7 weeks	33% in SBP (from 115 to 153 mmHg) [51]
Wistar rats	Ethanol 15%v/4 weeks	23% in SBP (from 119 to 147 mmHg) [52]

* In all cases, studies were conducted in male rats/mice; (*v*/*v*): volume ratio; MAP: mean arterial pressure; SBP: systolic blood pressure.

## Data Availability

Not applicable.
